# Using linked administrative data to describe a cohort of young people who have a parent with a history of homelessness (Study 1)

**DOI:** 10.23889/ijpds.v9i5.2697

**Published:** 2024-09-18

**Authors:** Jino Distasio, Aynslie Hinds, Corinne Isaak, Jaime Cidro, Nathan Nickel, Sarah Zell, Jitender Sareen

**Affiliations:** 1 University of Winnipeg; 2 University of Manitoba

## Objective and Approach

Little is known about the children of parents who experience homelessness. The At Home/Chez Soi (AHCS) Research Demonstration Project, launched in 2009, was a Canadian multi-site, multi-year study that tested the effectiveness of Housing First. In Winnipeg, 513 people were enrolled, of whom 47% were parents. Our objective was to determine how the children were faring with respect to their health and social situations at a time their parent was experiencing homelessness.

## Approach

The cohort, created using the Manitoba Centre for Health Policy’s familial linkage methodology, consisted of 405 individuals (< 30 years old) who were the offspring of the Winnipeg AHCS cohort. We descriptively summarized the socioeconomic characteristics and indicators of their involvement (or lack thereof) in various government systems at or in a period up to their parent’s study enrolment date.

## Results

Approximately half of the cohort were female (51%) and under 13 years (52%). Most (76%) resided in a household that had received income assistance. At least 50% had been placed into care at or near birth and only 17% had not been involved with the child welfare system. Of those school aged, 81% were enrolled in school and of those older than school aged, 53% had graduated. Nearly half (49%) had been diagnosed with a mental health disorder and/or with asthma (47%).

## Conclusions and Implications

This work may direct future research and may be used to guide health services policy changes and improve outcomes after prison release.

